# Protein quality control meets transcriptome remodeling under stress

**DOI:** 10.15698/cst2017.12.115

**Published:** 2017-11-15

**Authors:** Veena Mathew, Peter C. Stirling

**Affiliations:** 1 Terry Fox Laboratory, British Columbia Cancer Agency. 675 West 10th Avenue, Vancouver, British Columbia, V5Z1L3, Canada.

**Keywords:** splicing, genotoxic stress, ribosome production, protein aggregate, Saccharomyces cerevisiae

## Abstract

To tolerate and recover from genotoxic stress cells must coordinate a range of stress response activities including cell cycle arrest, DNA repair, and remodeling of the transcriptome and proteome. The suppression of ribosome production is a key feature of many stress responses in yeast, and much is known about the dynamics of this process at the transcriptional level. In our recent study, (J Cell Biol doi: 10.1083/ jcb.201612018) we focus on the stress related dynamic behaviour of a splicing factor called Hsh155, which is a core component of the SF3B subcomplex of the U2 small nuclear ribonucleoprotein complex, homologous to human SF3B1. The disassembly from its complex and sequestration of Hsh155 into nuclear protein aggregates contributes to suppressing ribosome production post-transcriptionally by promoting intron retention in ribosomal protein gene transcripts. The relocalization of Hsh155 is facilitated by TORC1-driven transcriptional changes and molecular chaperones that recognize disassembled Hsh155, eventually aiding in efficient recovery from stress.

Protein quality control (PQC) is an essential part of cellular homeostasis and encompasses the balance of non-native protein folding and degradation. Under stress, triage of non-native or disassembled proteins to aggregate structures mitigates cellular damage and allows cells time to either degrade or reactivate aggregated proteins upon stress recovery. We identified Hsh155 in a screen for GFP-fused proteins that moves after treatment of yeast cells with methyl methanesulfonate, hydrogen peroxide or ultraviolet light. Hsh155 moves into foci after MMS treatment and disassembles from its partners Hsh49 and Cus1 in the SF3B complex. This indicated to us that Hsh155 becomes non-native after stress. Accordingly, we found that it localized with markers of a recently identified nuclear PQC compartment called the intranuclear quality control (INQ) compartment. We tested the dynamics of Hsh155 in its aggregated form and found a shift of aggregate accumulation from INQ to cytoplasm over time, leading to a younger pool in the cytoplasm. As with other PQC localized proteins, Hsh155 sequestration is promoted by the aggregases Btn2 and Hsp42, and counteracted by disaggregases like Hsp104. Loss of Hsh155 function or loss of the aggregase Btn2 impaired the recovery of cells from MMS treatment upon stress removal. Interestingly, Hsh155 localization was not influenced by loss of the canonical DNA damage signaling kinases Tel1 and Mec1, the yeast orthologues of human ATM and ATR, suggesting that other signals were likely stimulating the disassembly and aggregation of Hsh155.

Whole proteome analysis of MMS treated cells confirmed suppression of ribosomal protein production, which had previously been seen as a major feature of transcriptional level change under different stresses. In yeast, ribosomal protein genes (RPGs) encode the vast majority of spliced transcripts due to the preponderance of introns in RPGs and their high expression levels. Thus, a shut-down of RPG transcription would dramatically reduce the need for splicing flux during stress. We observed the expected suppression of RPG expression after MMS treatment but saw a dramatic spike in intron-retention of RPG transcripts, suggesting that splicing was specifically inhibited. Remarkably, this splicing inhibition was dependent on the aggregase Btn2, as *btn2*Δ cells failed to increase intron retention in MMS. Together with our data showing that Btn2 is important for efficient stress recovery, this suggests that post-transcriptional shutdown of splicing may be a regulat-ed part of the stress response, which enforces suppression of RPG expression coupled with Hsh155 sequestration during stress.

Finally, we wondered whether transcriptional repression was a pre-requisite to Hsh155 disassembly and relocalization to INQ. It was previously known that TORC1 signaling is important to control the dynamic responses of RPGs to stress through a transcription factor called Sfp1. Sfp1 rapidly relocalizes to the cytoplasm in a TORC1-dependent manner under stress, and this facilitates the precipitous drop in RPG expression. Accordingly, we found that treatment of cells with the TORC1 inhibitor rapamycin or genetic perturbations of TORC1 pathway components suppressed Hsh155 movement to INQ. Indeed, deletion of the *SFP1* gene completely blocked Hsh155 localization to INQ in MMS. Thus, we created a model in which stress signaling through TORC1, leads to the eviction of Sfp1 from RPGs, their transcriptional repression and liberation of spliceosomes from chromatin. The SF3B complex then disassembles and Hsh155 is sequestered by molecular chaperones at INQ until stress subsides (**Figure 1**).

**Figure 1:**
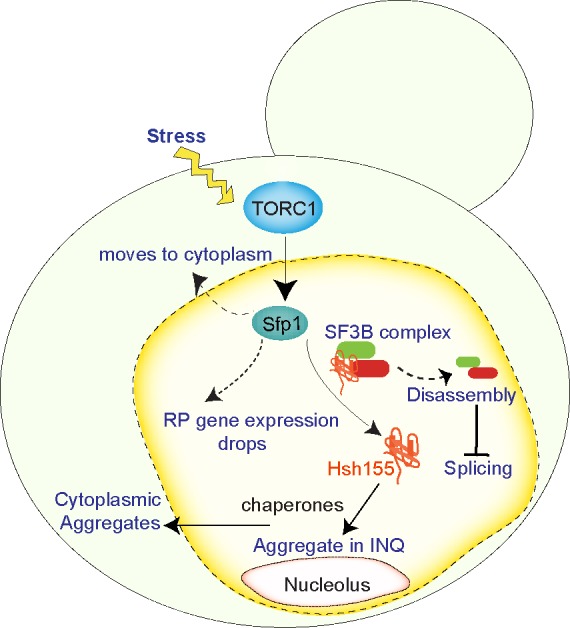
FIGURE 1: Schematic of a yeast cell illustrating our results which link stress induced TORC1-regulated gene expression changes to SF3B complex disassembly and Hsh155 recruitment to peri-nucleolar, and then cytoplasmic, aggregates. This process contributes to ribosomal protein (RP) gene expression decreases and inhibition of splicing under stress.

Interestingly, two other INQ substrates, Cmr1 and Hos2, are known to bind to RPG promoters and are proposed as transcriptional regulators. Thus, a bigger picture emerges in which eviction of several proteins from chromatin due to transcriptional remodeling under stress may require protein aggregate sequestration for optimal stress tolerance. The potential for regulated coordination of transcriptome remodeling with protein sequestration in aggregates is one of the most exciting phenomena suggested by our work.

One of the most obvious unanswered questions is why Hsh155, and no other proteins from the SF3B complex or the spliceosome relocalize to the INQ. What is special about Hsh155, Cmr1, Hos2 and other INQ resident proteins that makes them susceptible to genotoxic stress and why are they sequestered by the cell, rather than degraded? Careful analysis of INQ composition under different conditions should help to elucidate the common features of INQ substrate proteins under stress and may give insight to the signals driving their aggregation. Our work also sheds light towards broader questions about the nature and conservation of nuclear PQC. INQ forms adjacent to the nucleolus but what nuclear landmarks might dictate this positioning at the molecular level are unknown. Cells must coordinate the suppression of ribosomal RNA (rRNA) and RPG transcripts and it is tempting to speculate, based on the proximity of INQ to the nucleolus, that there may be some influence of INQ formation on rRNA biogenesis. Finally, potential links to human nuclear PQC are currently unclear. Human cells in culture do sequester proteins in the nucleolus under stress, providing a potential link to INQ behaviour that merits future study. Together, future studies looking at the molecular composition of INQ, how specific substrates are recruited, and how INQ composition determines global cell stress tolerance and survival, should reveal significant new insights to transcriptome dynamics in the stress response.

